# Topohistological alignments of ocular/penile organs

**DOI:** 10.1007/s12565-025-00844-3

**Published:** 2025-05-13

**Authors:** Gen Yamada, Daiki Hashimoto, Kota Fujimoto, Masanori Nakata, Shinichi Asamura, Yasuhiko Kawakami, Peter Lwigale

**Affiliations:** 1 Department of Plastic and Reconstructive Surgery, Wakayama Medical University, Wakayama, Japan; 2 Department of Physiology, Faculty of Medicine, Wakayama Medical University, Wakayama, Japan; 3 Department of Urology, Urological Science Institute, Yonsei University College of Medicine, Seoul, South Korea; 4 Department of Genetics, Cell Biology and Development, University of Minnesota, Minneapolis, MN 55455 USA; 5 Department of Biosciences, Rice University, Houston, TX 77005 USA; 6 Department of Plastic Reconstructive Surgery and Developmental Genetics, Wakayama Medical university, Kimiidera 811-1, Wakayama City, Wakayama 641-8509 Japan

**Keywords:** Topohistology, Eye, Penis, Vasculature, Pressure

## Abstract

Mammalian visual and genital (hereafter mainly penile) organs have been extensively studied albeit separately. Both organ systems contain sensation devices necessary for visual perception and sexual intercourse. Their terminal structures are covered with eyelid/prepuce followed by the sensitive epithelia of cornea/glans facing the eyeball and glans. These structures have been closely studied in humans for appropriate visual perception and copulation and have thus been treated by numerous surgeries for long periods. Despite the vastly divergent anatomy and physiological functions, there are a few intriguing topohistological similarities for both structures, functions, and pathology. The current article focuses on such features from various viewpoints.

## Introduction

Humans have historically paid attention to the healthy, homeostatic conditions of visual/genital organs for the use of perception and reproduction. We have long performed surgeries on eyelids for cosmetics and anti-aging purposes, as well as performed circumcisions since ancient times, showing keen attention to functional improvement among these organs. Although the two organs play essential roles for different visual/reproductive functions, little has been studied on the few shared features. Recent works on the lymphatic marker for the ocular system in the Schlemm's canal as well as such expression in the penis have previously been published (Fujimoto et al. 2024; Schnabellehner et al. 2024). In addition, several works on corneal/glans epithelia and collagen expression in sclera/tunica were also published (Fernández-Trillo et al. 2020; Lam et al. 2023; Antoniassi et al. 2020). Considering such findings, the current manuscript discusses the “potential” similarities of topohistology between ocular and penile systems.

### Topohistological alignments for “covering” the ocular/penile organs

**Fig. 1:**
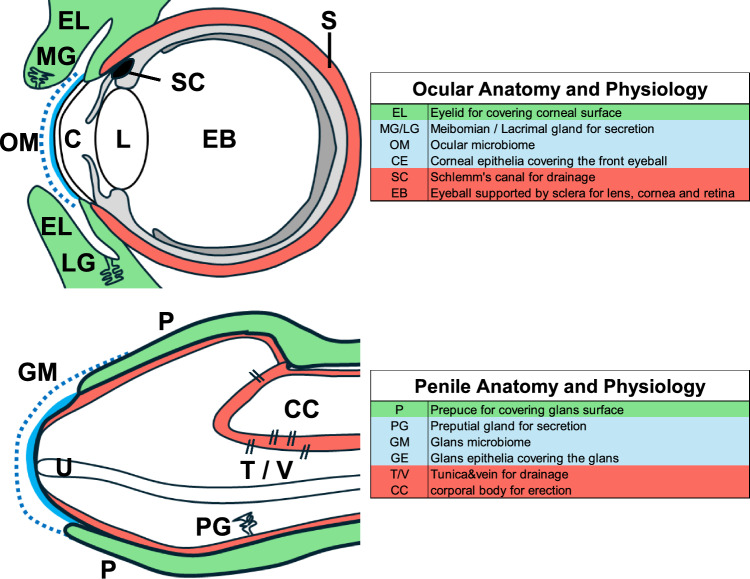
The coverage structure of the eyelid (EL) and prepuce (P) for the visual and penile systems (shown in green). Adjacent to such coverage, there locates sensitive epithelia containing the microbiome (ocular microbiome, OM and genital microbiome, GM) for corneal (C) and glans epithelia (shown in blue and dotted line). Each organ contains secretory glands, such as meibomian/lacrimal gland (MG/LG) and prepuce glands (PG). Adjacent to such structure, there locates solid and fibrinous collagen-rich pressure-regulated structures, eyeball sclera (S) and penile tunica albuginea and vein (T/V) (shown in red). Each organ contains tissues such as Schlemm's canal (SC) necessary for pressure drainage for the eyeball. In the case of the penis, there locates CC (corpus cavernosum) inside the penis with properly regulated pressure for erection/flaccid states. Lyve-1 positive character of sinusoids is reported adjacent to tunica, which might be related to drainage functions. The above topohistological landmarks are aligned with respective functions. The green color in the figure corresponds to the coverage structure, and the blue one indicates the epithelial structure and microbiome. Red parts show the fibrous structure. *U* urethra, *EB* eyeball

Anatomical structure, related physiological conditions, pathologies, and medical treatments are summarized in Fig. 1 and Table 1.

**Table 1:**
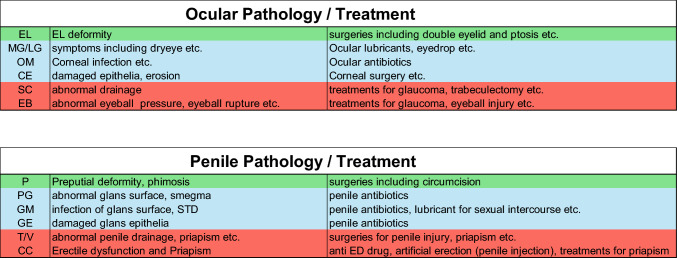
The table summarizes various pathologies and medical treatments for the two organ systems shown by the corresponding colors of Fig. 1, the coverages (eyelid and prepuce), secretary glands (Meibomian/Lacrimal gland and preputial glans), microbiome (ocular and glans), pressure-holding and drainage system (Schlemm's canal and tunica albuginea) and the fibrinous structure of eyeball sclera and tunica albuginea in CC

The terminal (distal) penis contains the prepuce (P) adjacent to the glans (G) region (Fig. 1, the green part) facing the penile shaft containing the erectile tissue with sinusoids. The prepuce functions to cover the surface of the glans and is exposed during the copulation for the glans epithelia surface. Such coverage is also included within the eyelids for the visual system (Fig. 1, the green part), followed by subsequent spaces of ocular and glans vestibules containing meibomian glands (MG) and lacrimal glands (LG) forming the ocular and penile microbiome by prepuce glands (PG) for secretary functions (blue dotted line; Fig. 1). Adjacent to them lies sensitive epithelia (cornea (Lwigale 2015) and glans; the blue line in Fig. 1). Healthy coverage and vestibular conditions are essential for visual perception and sexual intercourse to reduce friction during copulation. This is also related to reducing infection rates, including corneal bacteria and sexually transmitted diseases (STD). Excessive secretion often results in the accumulation of abnormal products. This includes eye mucus/discharges and penile smegma, both of which are related to several infections.

Both secretory functions are also affected by hormonal actions, namely estrogen and androgen, corresponding to female and male organs. Lower levels of estrogen/androgen due to aging effects reduce levels of MG/LG functions, often displaying dry eye in many females (Schaumberg et al. 2001; Krenzer et al. 2000; Esmaeli et al. 2000). Reduced levels of androgen, also due to aging, are known to induce lower PG functions, influencing the degree of sexual intercourse. The eyelid (EL), under the regulation of the peripheral nervous system, covers the cornea which is essential for the humid and smooth condition of its surface. Various pathological conditions are known to disrupt such coverage, including abnormal trigeminal nervous systems and defective secretory functions (Okada et al. 2019).

Furthermore, corneal and glans epithelia are particularly sensitive, with both expressing the sensory receptor from the PIEZO family. Such functions of sensation are essential for avoiding external injury of corneal epithelia and necessary for sexual satisfaction in the case of glans. Corneal PIEZO2 functions for mechanosensation, inducing the ocular blink for protective reactions (Fernández-Trillo et al. 2020). In case of glans (mainly reported for clitorial glans) and perineal region, PIEZO2 is also expressed for mechanosensation necessary for sexual satisfaction (Lam et al. 2023). Coverage of such epithelia is performed often by contact lenses to adjust visual perception as well as by condoms to protect against STD and ejaculation into the female reproductive tract (FRT). Contact lenses are mostly used for vision correction but also for protective coverage purposes post-surgically.

### Topohistology for regulating the inner pressure of eyeball/glans

The inner structure of each organ differs for holding ocular apparatus and erectile tissue. Of note is the gross anatomical alignment for both systems.

Figure 1 and Table 1 show comparative anatomy, pathophysiology and medical treatments of ocular organs and penis. Facing the coverage epithelia lies the solid fibrous collagen-rich structures (eyeball sclera and penile tunica albuginea; the red part in Fig. 1) supporting physiologically relevant pressure levels. Generally, the main components of such connective tissues include a solid, fibrous, collagen-rich structure as well as related pathological conditions, thus showing a few intriguing similarities.

The inner pressure-regulated morphology for the ocular eyeball/erected penile shape is necessary for each physiological function. In the case of eyeballs, such pressure is essential for keeping their constant homeostatic volume. This results in a sphere-shaped ocular eyeball that is established by such pressure (intraocular pressure, IOP) inside the sclera. The dysregulation of such inner ocular pressure is further associated with abnormal corneal pathologies. Elevated pressure leads to protruded eyeballs associated with dry eye, which is known for the case of Graves' disease (Gamblin et al. 1983).

Likewise, penile erected status is achieved by pressure-regulated sinusoidal expansion (intracovernosal pressure, ICP) inside the solid fibrous tunica region in contrast to the normal (flaccid) state. Higher erectile pressure is linked to the conditional changes of prepuce and glans epithelia. During erection and copulation, the prepuce shows retraction with the exposed glans epithelia, which should be covered with a moist fluid by PG and also by vaginal secretory functions. Abnormality of such moist conditions leads to sexual dysfunction and often dyspareunia in females.

Damages in such pressure regulation should thus be avoided. Rupture and disruption of the ocular sclera and penile tunica region are both caused by external injury, which induces critical damages to both pressure regulations. Such injuries should be immediately treated by the corresponding surgery. Modulation of the inner pressure could also be performed through medical treatments. Gas injection can be performed for visual abnormalities, including retinal detachment. Furthermore, artificial gel or similar substances can be injected into the erectile tissue to assist erectile functions.

Aqueous humor, included inside the sclera, is transparent fluid, and its composition is often described as similar to that of lymphatic fluid, one of the common tissue fluids (Goel et al. 2010; Benagiano et al. 2024). In the case of the penis, the fulling and erection state is achieved through blood and it’s change in pressure. Of note, it was reported that lymphatic fluid is responsible for erection in some species of aves (Yamano et al. 1977; Budras and Berens von Rautenfeld 1978). In such species, the erectile tissue is thus termed as corpus para-cloacalis vascularis with a lymphatic spongy body showing one type of erectile fulfillment. Such erection is generally “weaker” as lymph is basically a low-pressure system when compared to blood (Brennan 2022).

Because of the tightly shield structure, it has been known that the ocular chamber, filled with aqueous humor, is a sort of immune-privileged region allowing transplantation of a cornea for treatment. In the case of the penis, the genitalia is frequently exposed to STDs as it faces foreign bodies through sexual intercourse. With this, glans surface or urethra is frequently infected by STDs. It is also known that the erectile tissue, the sinusoid, is the region with less-frequent infection. Thus, both organ systems could be stated to function in a vastly diverged manner albeit with a few similarities. Of note are recent reports on the possibly necessary structures for the outflow regulation. The pressure is regulated by the inflow and outflow, which is, in part, achieved by Schlemm's canal in the ocular system and the tunica/venous (T/V) region for erectile tissues. Lyve-1, the well-known lymphatic marker, is expressed in Schlemm's canal in the ocular drainage system (Park et al. 2014). In the case of penile outflow regulation, the essential role of tunica albuginea and its associated vein is known. Lyve-1 is expressed rather closely to the tunica albuginea region, and its significance in penile outflow still requires investigation (Fujimoto et al. 2024; Schnabellehner et al. 2024). The structures of Schlemm's canal adjacent to the trabecular mesh region and vasculature in the tunica albuginea region are considered to regulate the drainage by pressure-induced compression. In such aspects, the compressive regulation for both vasculatures is mediated by the surrounding fibrous connective mesenchyme, and further studies are necessary to reveal their precise roles.

### Few, albeit notable, symptoms shared in the two diverged vasculatures

Ocular and penile systems develop complex vasculatures necessary to support their different functions. In the case of the ocular system, complex retinal vasculatures constitute an essential part of visual perception. In contrast, penile sinusoids in the corpus cavernousum are essential vascular components, displaying their contraction/relaxation. Reflecting their vastly different functions, retina and sinusoids play essential roles in visual perception and erection for sexual intercourse. Albeit with such divergence, there are a few shared symptoms for the two vascularized organs.

Generally, the intra-tubular blood-derived pressure prominently affects the development and the pathogenesis of vasculatures, including endothelial cells and surrounding smooth muscle. During development, vasculature-induced organogenesis is known, and the dysregulated pressure leads to abnormalities of the endothelium and surrounding smooth muscle layers. Retina and penile sinusoids are both exposed prominently to pressure of vascular blood. Because of this, highly developed vasculatures supply nutrients to visual perception units, and such vasculatures are sensitive to high blood pressure. The retina is a sensitive tissue of vascular damage derived from high blood pressure and diabetes, and its associated retinopathy is a serious health issue as of recently (Sinclair and Schwartz 2019; Chew et al. 2013). Erection is mediated by the regulation of contraction/expansion of sinusoids, and erectile dysfunction (ED) is one of the common vascular abnormalities in men’s health. The penile sinusoids are also exposed to high blood pressure with the dramatic change into a flaccid state. Continuously, high-pressure (erection) is termed priapism, and the lower one leads to ED for sexual intercourse. Of note is the clinical association of some ED and diabetic retinopathy (Henis et al. 2011; Chew et al. 2013). Measurement of retinal vessel caliber shows the association of impaired retinal vessels and ED patients with diabetes (Chew et al. 2013), and drugs for ED and other penile diseases should also be considered for their effects on the retina and ocular abnormalities (Polak et al. 2003).

During the wound-healing processes, fibrosis is often observed depending on the abnormal processes. Several forms of fibrosis are observed after ocular and penile tissue damages, and it is often observed concordant with endothelial dysfunction and repair. They include the ectopic chondrogenesis in the case of murine penis (Hashimoto et al. 2021), and retinal fibrosis is often reported along its inner surface.

## Conclusion

Ocular and penile functions as well as their pathophysiologies have been independently studied and analyzed. With this, wide spectrum and broadly interdisciplinary viewpoints are required to further analyze and treat both organ systems.
